# Identification of genetic susceptibility in preterm newborns with bronchopulmonary dysplasia by whole-exome sequencing: BIVM gene may play a role

**DOI:** 10.1007/s00431-022-04779-z

**Published:** 2023-02-09

**Authors:** Xi Luo, Min Zhao, Cheng Chen, Fengji Lin, Xiaodong Li, Haiyun Huang, Lei Dou, Jinxing Feng, Shanqiu Xiao, Dong Liu, Junli He, Jialin Yu

**Affiliations:** 1 Department of Neonatology, Children’s Hospital of Chongqing Medical University, National Clinical Research Center for Child Health and Disorders, Ministry of Education Key Laboratory of Child Development and Disorders, Chongqing Key Laboratory of Child Infection and Immunity, Chongqing Key Laboratory of Pediatrics, 136 Zhongshan 2nd Road, Yuzhong District, Chongqing, 40014 China; 2 Department of Neonatology, Southern University of Science and Technology Hospital, No. 6019 Liuxian Avenue, Xili Street, Nanshan District, Shenzhen, 518055 China; 3 Department of Neonatology, Shenzhen Longgang District Maternity & Child Healthcare Hospital, Shenzhen, 518172 China; 4 Department of Neonatology, Huazhong University of Science and Technology Union Shenzhen Hospital (NanShan Hospital), Shenzhen, 518052 China; 5 Department of Neonatology, Shenzhen Children’s Hospital, Shenzhen, 518031 China; 6 Department of Neonatology, Shenzhen Baoan Women’s and Children’s Hospital, Shenzhen, 518133 China; 7 Department of Neonatology, Shenzhen People’s Hospital, Shenzhen, 518020 China; 8 Department of Neonatology, Shenzhen University General Hospital, Shenzhen, 518055 China

**Keywords:** Bronchopulmonary dysplasia, Genetic susceptibility, Whole-exome sequencing, Single-nucleotide polymorphisms, BIVM gene

## Abstract

Bronchopulmonary dysplasia (BPD) is a common chronic respiratory disease in preterm infants caused by multifactorial etiology. Genetic factors are involved in the occurrence of BPD, but studies have found that candidate genes have poor reproducibility and are influenced by ethnic heterogeneity; therefore, more exploration is still needed. We performed whole-exon sequencing in 34 preterm infants with BPD and 32 non-BPD control neonates. The data were analyzed and interpreted by Fisher difference comparison, PLINK and eQTL association analysis, KEGG and GO enrichment analysis, STRING tool, Cytoscape software, ProtParam tool, HOPE online software, and GEOR2 analysis on NCBI GEO dataset. BPD has a highly heterogeneity in different populations, and we found 35 genes overlapped with previous whole-exon sequencing studies, such as APOB gene. Arterial and epithelial cell development and energy metabolism pathways affect BPD. In this study, 24 key genes were identified, and BIVM rs3825519 mutation leads to prolonged assisted ventilation in patients with BPD. A novel DDAH1 mutation site (NM_012137: exon1: c.89 T > G: p.L30R) was found in 9 BPD patients.

*Conclusion*: BIVM gene rs3825519 mutation may play a role in the pathogenesis of BPD by affecting cilia movement, and the DDAH1 and APOB genes mutations may have a pathogenic role in BPD.

**Table:** 

**What is Known:**
• *Genetic factors are involved in the occurrence of bronchopulmonary dysplasia.*
• *The candidate genes have poor reproducibility and are influenced by ethnic heterogeneity, therefore, more exploration is still needed.*
**What is New:**
• *We identified the role of susceptible SNPs in BPD in Shenzhen, China, and identified 24 key genes that influence the pathogenesis of BPD, and also found 35 genes overlapped with previous whole exon sequencing studies, such as APOB gene.*
• *We found that BIVM and DDAH1 genes may play a pathogenic role in the pathogenesis of BPD.*

**Supplementary Information:**

The online version contains supplementary material available at 10.1007/s00431-022-04779-z.

## Introduction

Bronchopulmonary dysplasia (BPD) is a common chronic respiratory disease in premature infants caused by complicated pathogenesis. In recent years, the survival rate of preterm newborns is increasing; however, the incidence of BPD is also increasing. In the USA, 63% of extremely preterm infants (EPI) are complicated with BPD [1], which has become the leading cause of death in preterm infants after 60 days [2]. In China, the incidence of BPD in EPI increased from 19.3 to 51.7% from 2010 to 2017 [3, 4]. Furthermore, BPD will cause a series of complications, such as airway hyperresponsive disease and recurrent infections in lower respiratory tract, which brings a heavy burden on society and families. Finding ways of early diagnosis for BPD is particularly important and urgent.

In twin studies, genetic factors were found to account for 53–82% of susceptibility to BPD [5, 6]. Numerous studies have attempted to identify the key genes involved in BPD; however, due to many reasons, the reproducibility of the candidate genes is poor. Whole-exon sequencing (WES), as a means of gene sequencing, can be used to explore common and rare coding gene mutations that may directly affect protein structure and function. There were five studies using WES to identify BPD mutations [7–11] included infants in Italy, the USA, France, and Shanghai in China. Considering the differences in race and the research in Shanghai was a single-center study, it is necessary to validate the candidate genes in these five studies and to explore whether there are new key genes of BPD in Chinese preterm newborns.

## Materials and methods

### Participants

We recruited 34 BPD patients and 32 non-BPD infants from 7 hospitals in Shenzhen from January 2020 to May 2022. The diagnostic criteria for BPD are based on the new consensus at 2018 NICHD Symposium [12]: preterm infants (gestational age (GA) ≤ 32 weeks) at 36 weeks of postmenstrual age were still dependent on FiO_2_ and respiratory support ≥ 3 days, and had interstitial lung disease (image confirmation). The exclusion criteria were as follows: (1) congenital inherited diseases and malformations, (2) lost to follow-up. Non-BPD premature infants who hospitalized in the same period and did not have BPD were selected. The clinical characteristics such as GA, feeding mode, day age, and birth mode were matched with the BPD group. The oral mucosal epithelium of participants and clinical data were collected. The study was approved by the Ethics Committee of Shenzhen University General Hospital (Approval No. 2020–001-02), and the Chinese Clinical Trial Registration (Registration No. ChiCTR2000033610) was completed. All participants’ guardians signed informed consent.

### Exome sequencing

DNA were extracted by magnetic universal genomic DNA kit (TIANGEN DP705, Beijing, China), detected by Agilent 5400 fragments analyzer system (NYSE: A, California, USA), randomly interrupted by Covaris crusher (M220, Massachusetts, USA), purified by AMpure XP Reagent (Beckman A63881, California, USA). Library concentration was determined by fluorometer (Qubit 2.0, Thermo Fisher, USA). Exons were captured using the SureSelect Human All Exon V6 (Agilent Technologies, 5190–8865, California, USA) and streptavidin magnetic beads. The library was sequenced on NovaSeq 6000 platform (Illumina, California, USA). Sequencing reads quality was evaluated by FastQC package and removed the connector by cutadapt (v1.18), and discarded reads with N > 10% or the base number (the quality value *Q* ≤ 10) proportion > 50%; then, reads were uploaded to the CHI cloud analysis platform based on FANSe3 and were mapped to the human reference genome (GRCh37) to search mutations. Annovar software, hg19 refGene, the population frequencies of Exome Aggregation Consortium (ExAC, V.0.3.1), 1000g2015AUG and dbnsfp42a were used to annotate the mutation sites.

### Data analysis

Principal component analysis was used to detect outliers and layered of group [13]. Five software (SIFT, Polyphen2_HDIV, Polyphen2_HVAR, Mutation Taster, and PROVEAN) were used to predict the harmfulness of mutations. Deleterious mutations were considered only if at least three software predictions were harmful. PLINK2 (v2.00a3lm) software was used to analyze the association between mutation sites and disease. Three Gene Ontology (GO) analysis, Kyoto Encyclopedia of Genes, and Genomes (KEGG) pathway enrichment analysis were used for the screened variant genes. Venn diagram was used to show coincidence genes with previous literature [14]. Gene expression and quantitative trait loci (eQTL) analysis used linear regression models to assess associations between the single-nucleotide polymorphisms (SNPs) and gene expression in 500-kbs upstream and downstream of those SNPs, and the data used in eQTL analysis were downloaded from the Genotype-Tissue Expression (GTEx) data portal. The SNP-set (sequence) kernel association test (SKAT-O) for R-package SKAT was used to obtain the association between mutations in a gene and disease.

### Protein interaction network construction and protein structure prediction

We used STRING (Search Tool for the Retrieval of Interacting Genes Database, Version 11.5, https://string-db.org) tool to predict the relationship between different genes. Cytoscape software (Version 3.10.0) was used to visualize and analyze the interaction network. Degree ≥ 20 was set as the core gene. We used ProtParam tool (https://web.expasy.org/protparam/) to calculate the physical and chemical parameters of protein, which based on protein sequences given in NCBI. We used HOPE online software (https://www3.cmbi.umcn.nl/hope) to analyze the structure of the mutations.

### Expression of the mutated gene in the patient

We downloaded previously published sequencing data from the NCBI GEO dataset (http://www.ncbi.nlm.nih.gov/gds). The GSE32472 dataset was from the peripheral blood of premature infants with GA ≤ 32 weeks and birth weight (BW) < 1500 g. There were 68 BPD patients and 43 non-BPD infants. The GSE188944 dataset is sequencing data from umbilical cord blood of BPD patients (*n* = 6) and preterm infants without BPD (*n* = 17) in Argentina (GA ≤ 35 weeks and BW < 1500 g). After GEOR2 analysis, *P* < 0.05 and |logFC|> 1 were screened to obtain differentially expressed genes, which were used to find the overlapping genes with the candidate genes in this study.

### Statistical analyses

Approximately normally distributed data were described using mean ± standard deviation (SD) and evaluated using *T*-test analysis. Non-normally distributed data were described using medians and interquartile range (IQR) and evaluated using Fisher’s exact test. *P* values were adjusted using the Benjamini–Hochberg false discovery rate (FDR-BH), and *P* < 0.05 was considered statistically significant.

## Results

### Clinical characteristics of the patient

**Fig. 1:**
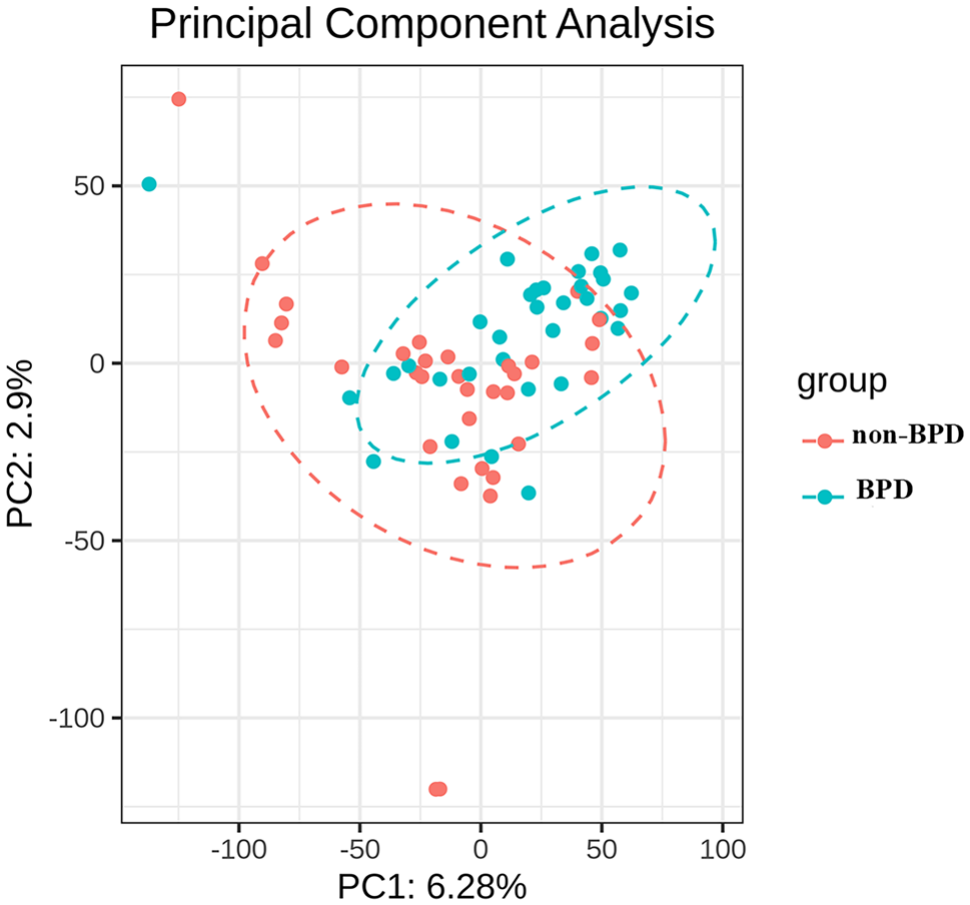
PCA plot of association test statistics in the WES-based discovery data

There were 34 BPD infants and 32 in non-BPD infants in the study. BPD patients’ GA and BW were statistical smaller and lighter than non-BPD infants, but there were no differences in gender, singleton, and multiple birth composition (Table 1). And, the results of principal component analysis showed there was no inherent diversity between these two groups (Fig. 1).

**Table 1:** Clinical characteristics of 66 premature infants

Characteristic	BPD (*n* = 34)	No BPD (*n* = 32)	*P*-value
Gestational age (week, mean ± S.D.)	27.6 ± 2.1	28.8 ± 1.9	0.021
Birth weight (g, mean ± S.D.)	948.5 ± 200.0	1206.2 ± 299.2	0.0002
Gender (%)			0.988
Boy	18 (52.9%)	17 (53.1%)	
Girl	16 (47.1%)	15 (46.9%)	
Multiple births, no.(%)	12 (35.3%)	10 (31.5%)	0.728
BPD grades (%)			
I	21 (61.8%)		
II	7 (20.6%)		
III	4 (11.8%)		
IIIA	2 (5.9%)

### Preliminary findings from WES data

**Fig. 2:**
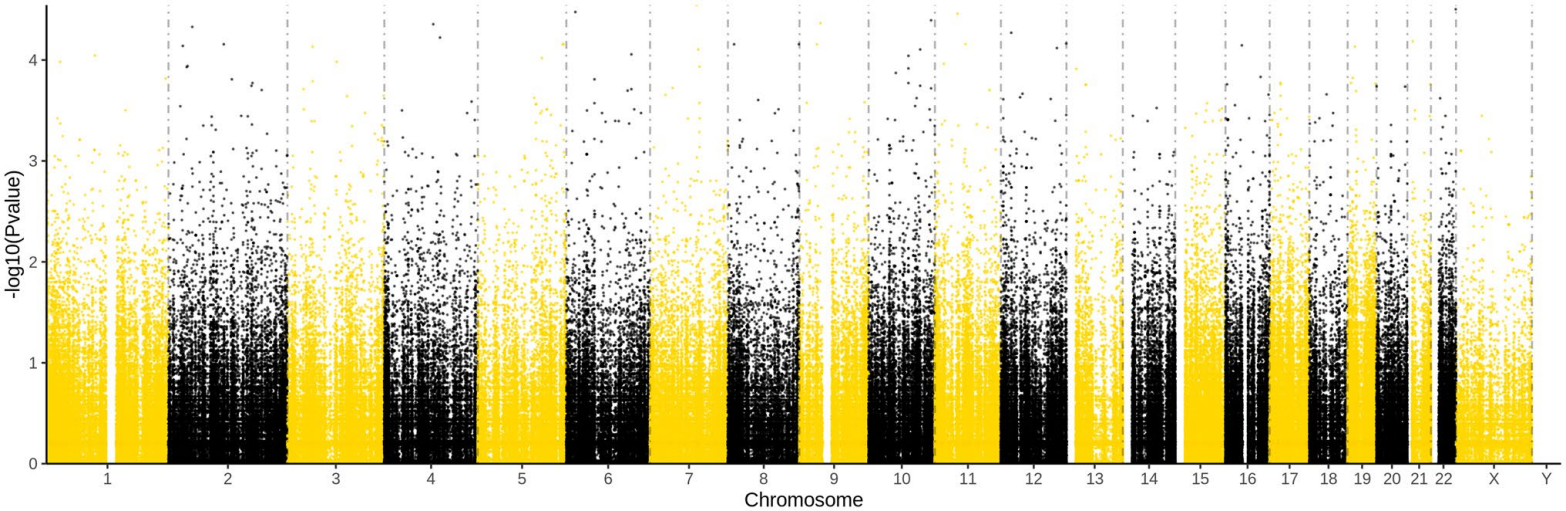
Manhattan plot of results from exome-wide association analysis of 34 BPD and 32 non-BPD infants

After stratification and annotation of gene mutation, there were 861,620 gene mutations (Fig. 2) and 27,745 were deleterious mutations. We compared the mutated genes between the two groups by Fisher’s exact test and screened out 457 differential SNPs, of which 336 (73.5%) were substitution mutations, 86(18.8%) were deletion mutations, and 37 (8.1%) were insertion mutations. Most of those SNPs were heterozygous mutations.

### Few gene mutations consistent with previous research

**Fig. 3:**
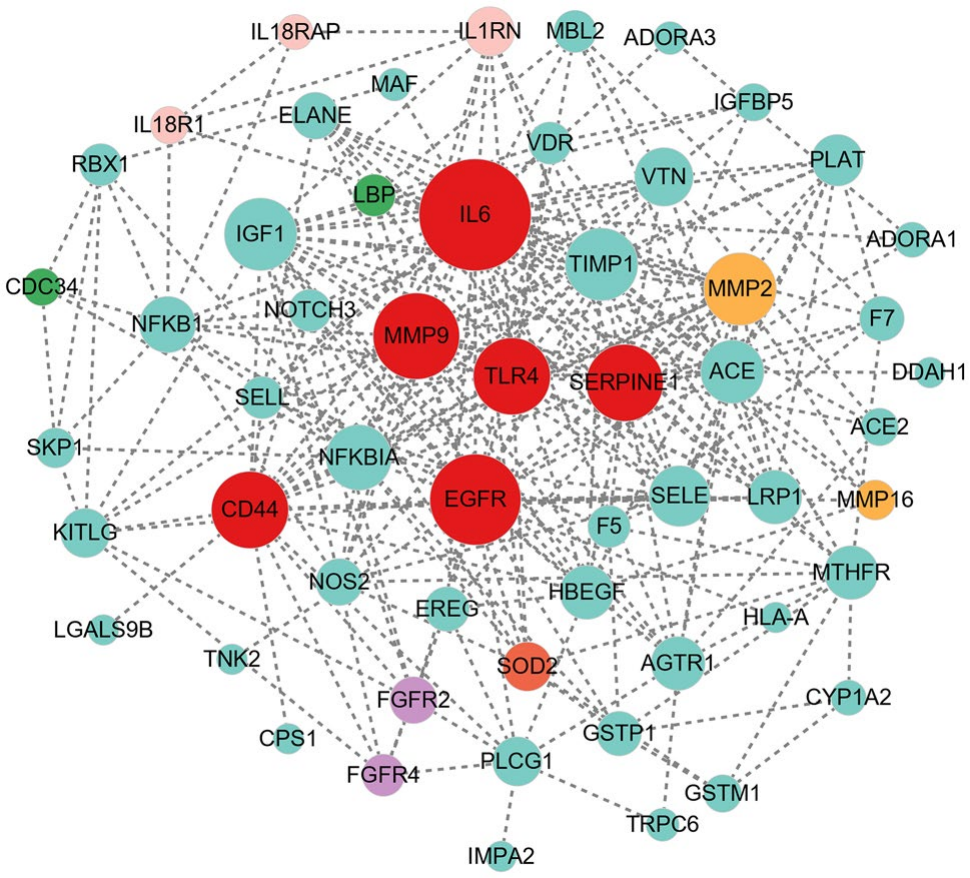
The mutant genes in our study were overlapped with those in previous studies. The genes with red color are core genes

In order to explore whether there are SNPs in line with previous reports, we searched databases (Phenopedia, DisGeNet, MalaCards, and GWAS catalog), and found 50 genes that have been studied for the relationship between SNP and BPD. There are 128 mutation sites in these 50 genes in our study (Supplementary Table 1). We through the interaction network to find the core genes were IL6, EGFR, MMP9, CD44, SERPINE1, and TLR4, which are inflammatory mediators, epidermal growth factor receptors, matrix metalloproteinase families, cell adhesion molecules, serine protease inhibitors, and toll-like receptors respectively (Fig. 3). In our study, IL6 (rs2069832, rs1474347), EGFR (rs11506105, rs17336919) and MMP9 (rs3787268), CD44 (rs34986068, rs7639388, rs3215691), and SERPINE1 (rs2227692, rs2854236) SNPs were more common to non-BPD group, suggesting these SNPs are not risk factors of BPD (Supplementary Table 2).

**Fig. 4:**
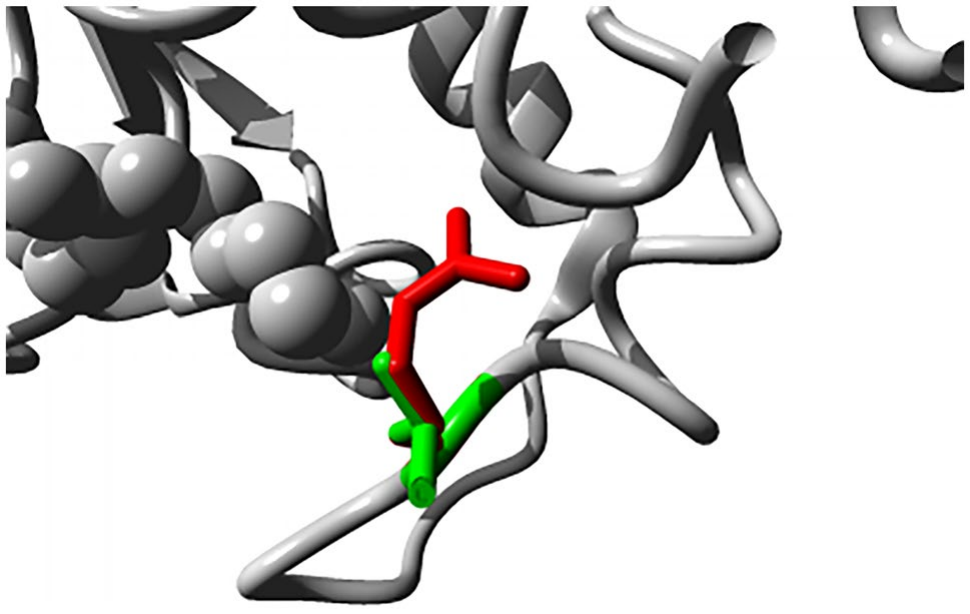
Predicted 3D structure of DDAH1 wild type and mutant. Close-up of the mutation. Wild-type, and mutant side chain are shown in green and red respectively, the rest of the protein is shown in gray

In this study, the SNPs found only in the BPD group were SOD2 (superoxide dismutase 2) rs5746091 (10 cases, 29%), rs5746090 (9 cases, 26%), rs28662077 (6 cases, 19%), and DDAH1 (NM_012137:exon1:c.89 T > G:p.L30R, 9 cases, 26%), of which SOD2 SNPs were in intron regions, and BPD patients with SOD2 rs5746091 mutation had a larger GA (*P* = 0.015, 28.9 ± 1.8 weeks vs 27.0 ± 1.9 weeks) and heavier BW (*P* = 0.002, 1108 ± 235.0 g vs 882.1 ± 136.5 g) than non-mutated BPD patients, BPD patients with SOD2 rs5746090 mutation had a heavier BW than non-mutated BPD patients (*P* = 0.01, 1093.3 ± 243.3 g vs 896.4 ± 151.0 g), suggesting those two SOD2 mutations may be related to child’s GA and BW. DDAH1 (dimethylarginine dimethylaminohydrolase 1, NM_012137: exon1: c.89 T > G: p.L30R) SNP is a non-synonymous mutation and located in exon region, whose residue larger and more hydrophilic than the wild-type and residual charge changes from neutral to positive (Table 2, Fig. 4). This mutation in DDAH1 was predicted to be a harmful mutation that easily leads to loss of interactions with the ligand, suggesting that mutations of this gene may play an important role in BPD.

**Table 2:** Changes in physicochemical parameters between mutant and wild-type proteins in DDAH1

Gene	Amino acids(*n*)	Molecular weight	Theoretical pI	Estimated half-life	Instability index	Aliphatic index	Grand average of hydropathicity	Type
DDAH1	285	31,121.78	5.53	30 h	38.12 (stable)	95.79	−0.133	
DDAH1-variant	285	31,164.8	5.64	30 h	39.45 (stable)	94.42	−0.162

**Fig. 5:**
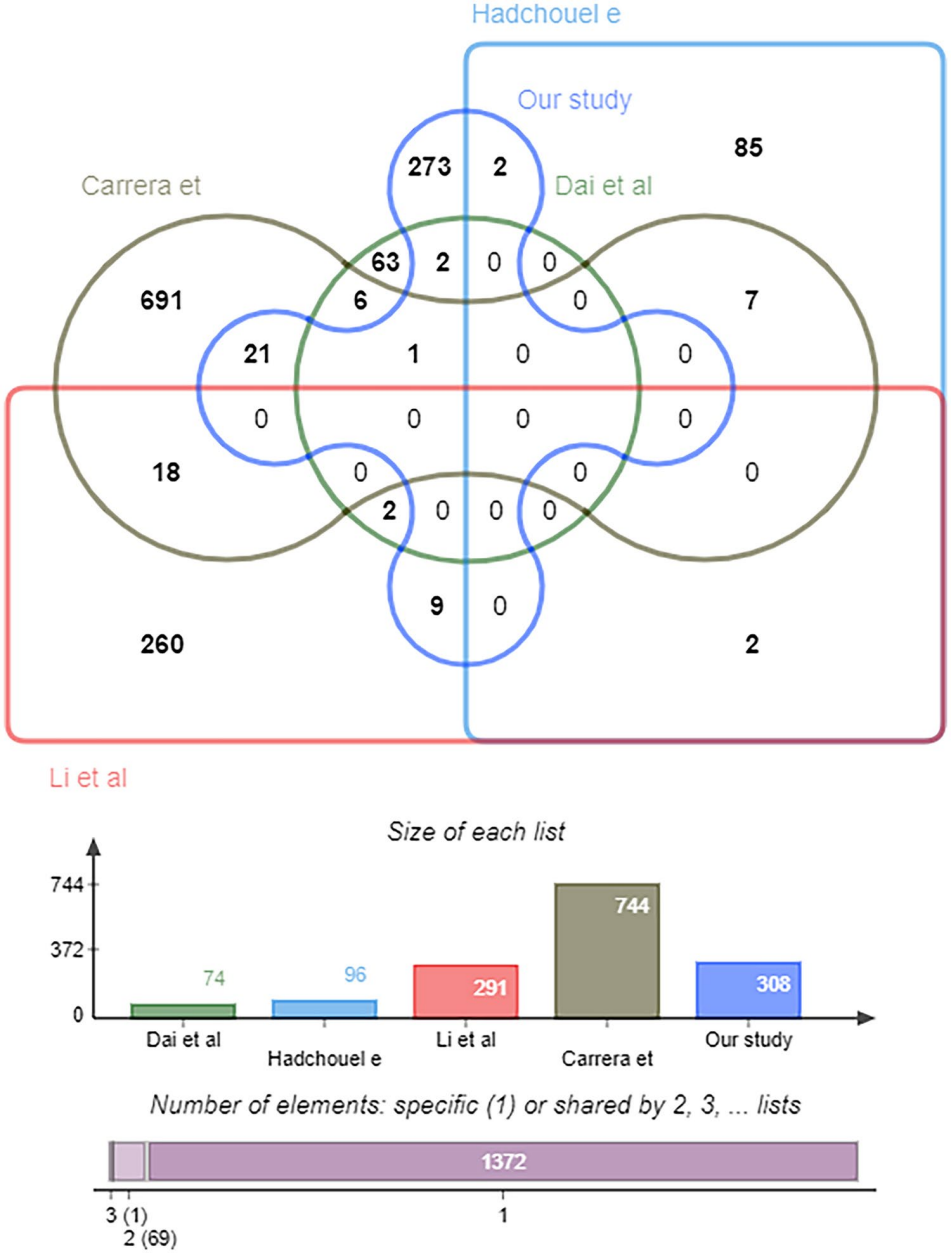
Venn diagram of risk genes for BPD development reported in four previous studies [16–19] and ours

Compared with previous WES studies, we found no common overlapping genes in all studies (Fig. 5), but 35 genes in our study have been reported in previous WES studies. There were three overlapping genes (GIGYF2, KRT10, APOB) with the study in Shanghai [10]. Twenty-two genes (MAP3K6, NCOR2, FAM155A, PAPLN, MYO9A, ABCC6, ABCC11, PIEZO1, DNAH2, MYO15A, RNF213, ACP5, APOB, CSPG5, FGFRL1, DNAH5, SOBP, ROS1, TBP, GLI3, TMEM229A, GALNTL5) overlapped with Carrera’s work [8], two genes (MAB21L3, LIFR) overlapped with Hadchouel’s [9] studies, and 9 genes (FIGN, ITGA9, ANKRD6, CNOT4, NAV2, CIT, SLC8A3, TRIP11, SETBP1) overlapped with study by Li [7]. Among them, only apolipoprotein B (APOB) gene was found in three studies, and these genes are mainly involved in cellular components (FAM155A, PAPLN, TMEM229A, GALNTL5, SLC8A3), immune-related (GLI3, SOBP), microtubule and ciliary organization (MYO9A, MYO15A, DNAH2, DNAH5, FIGN), angiogenesis (MAP3K6, RNF213), fibroblasts (FGFRL1), and WNT signaling pathway (ANKRD6), which indicate these pathways are important to BPD.

### Gene mutations strongly associated with BPD

**Fig. 6:**
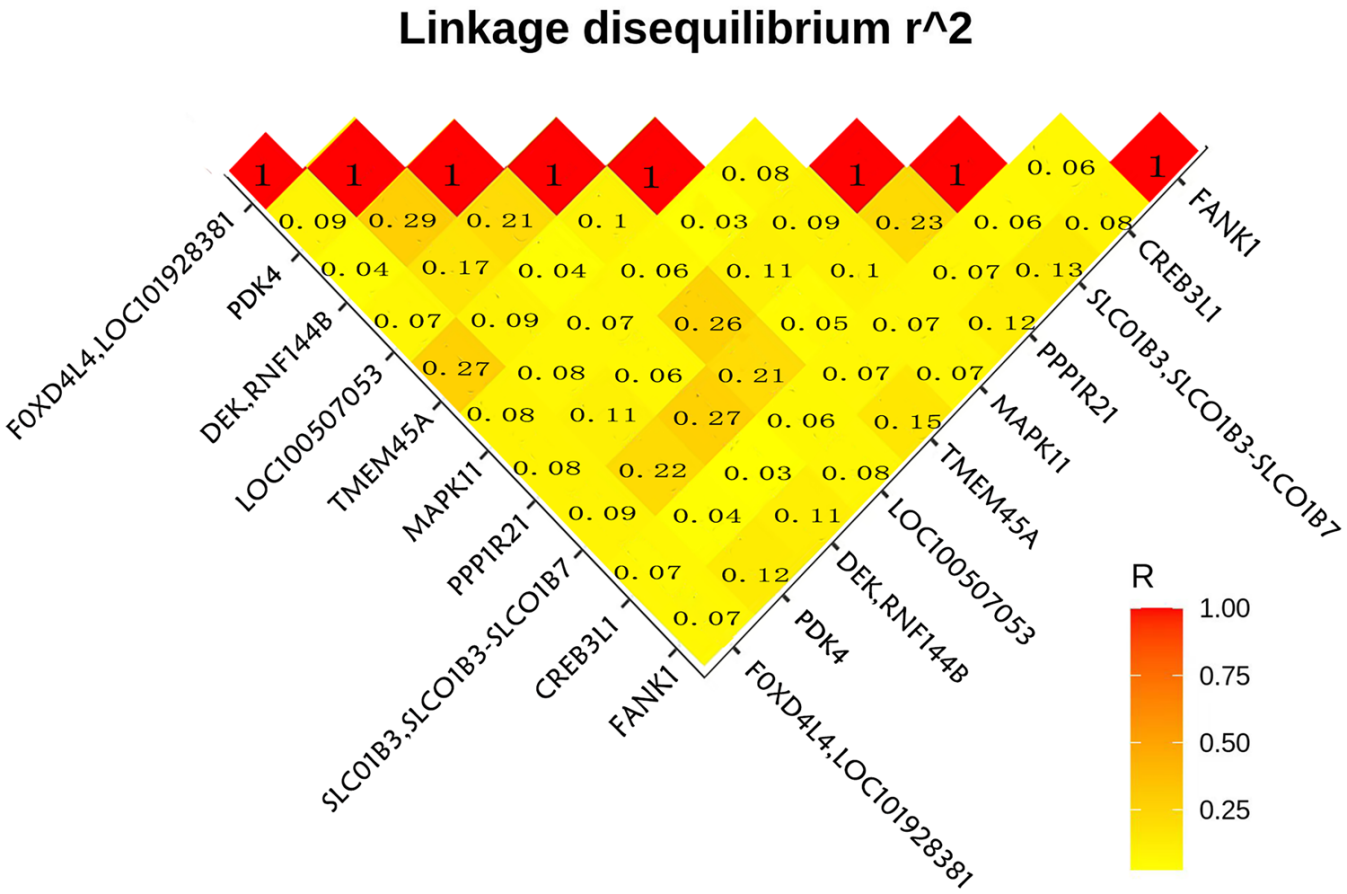
Linkage disequilibrium analysis among the top 10 SNPs associated with BPD

Association analysis of mutation sites and groups using PLINK2 revealed 19,256 SNPs associated with BPD, most of which were in the intron region. Only 5 SNPs had *P* value < 5 × 10^−5^ (Table 3), and no reports found they are related to BPD. Among them, DEK/RNF144B gene rs6928572 has a population carrier frequency of 91% in the 1000 genome database and a low pathogenicity. The harmfulness of SNP on CREB3L1 gene is not high in various databases. None of the top 10 SNPs was in linkage disequilibrium, suggesting that there is no common effect on these genes (Fig. 6).

**Table 3:** SNPs strongly associated with BPD in PLINK2 association analysis

ID	*P-*value	Region	Gene	rs
chr7_95215272_T_C	1.85E-05	intronic	PDK4	-
chr6_18364733_G_C	2.05E-05	intergenic	DEK, RNF144B	rs6928572
chr9_42752064_T_C	2.86E-05	intergenic	FOXD4L4, LOC101928381	rs62554164
chr22_50705729_A_T	4.42E-05	intronic	MAPK11	-
chr11_46329475_T_G	4.86E-05	exonic	CREB3L1	-

### Functional enrichment of the differential genes in two groups

**Fig. 7:**
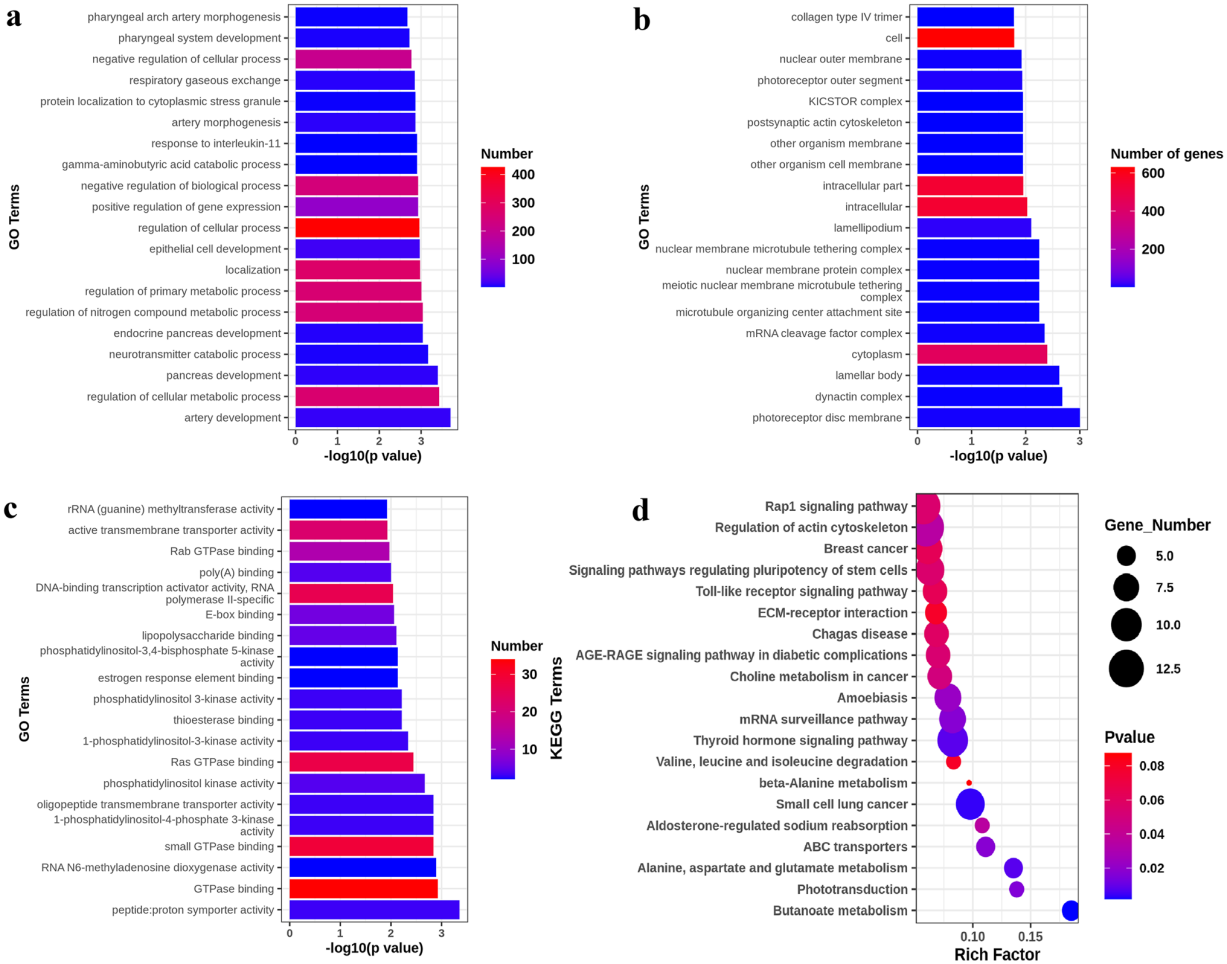
GO and KEGG enrichment analysis of differential genes associated with BPD. **a** GO enrichment analysis biological process, **b** GO enrichment analysis, cellular component, **c** GO enrichment analysis molecular function, d KEGG enrichment analysis

We found 3074 genes’ mutations were associated with BPD through SKAT-O test, and used GO- and KEGG-enriched pathways to find those genes in biological processes included arterial development, regulation of cellular metabolic processes, pancreas development, epithelial cell development, etc.; in cell composition, enriched pathways included photoreceptor disk membrane, dynactin complex, lamellar body, collagen type IV trimer, etc.; in molecular functions, enriched pathways included peptide, proton symporter activity, GTPase binding, RNA N6-methyladenosine dioxygenase activity, etc., suggesting that the mutated genes mainly cover blood vessels, epithelial cells, fibers, and energy metabolism. KEGG pathways are mainly involved in human diseases, metabolism, biological systems, etc. The top three are mainly methyl butanoate metabolism, phototransduction, alanine, aspartate, and glutamate metabolism (Fig. 7).

### Twenty-four candidate genes may influence the pathogenesis of BPD

To further understand the effect of gene mutations on surrounding genes, we performed eQLT analysis for gene mutations with *P* < 0.01 and found that 24 deleterious mutations affected the expression of surrounding genes, namely TMEM17, CTD-2521M24.6, DYRK4, KLHDC4, AC007362.3, CNIH2, CTSF, SFI1, NGDN, IFNAR1, PLD4, F8A1, PRELID1, TMX4, RP11-510M2.2, STRCP1, ICAM3, SNRNP70, WWC3, DNM1P51, BIVM, LIFR, CASP1P2, and SMG8 (Table 4), these genes were the candidate genes in the pathogenesis of BPD. To confirm whether these genes are differentially expressed in BPD patients, through the NCBI GEO dataset, we found decreased expression of BIVM (immunoglobulin-like variable motif) in the cord blood of BPD patients, and BIMV rs3825519 mutation was also found in our study; therefore, we speculated BIMV rs3825519 mutation may lead to decreased expression of BIMV gene.

**Table 4:** Twenty-four mutation-affected genes identified by eQLT analysis

Mutant gene	Region	rs	GTEx_related_gene	eQTL_*P*-value
TMEM17	Exonic	rs11676567	TMEM17	1.26E-08
MVB12A	Intronic	rs2303678	CTD-2521M24.6	8.10E-08
DYRK4	Intronic	rs2286575	DYRK4	2.92E-09
KLHDC4	Intronic	rs9934565	KLHDC4	4.01E-14
EEF1B2, SNORA41	Downstream	rs34451626	AC007362.3	0.000143
CTSF	Intronic	rs2242663	CNIH2	0.001544
CTSF	Intronic	rs2242663	CTSF	3.52E-24
DRG1	Intronic	rs1001599	SFI1	5.57E-23
NGDN	Exonic	rs2236261	NGDN	2.69E-17
IFNAR1	Intronic	rs2850015	IFNAR1	1.09E-15
AHNAK2	Exonic	rs34499888	PLD4	0.000103
TMLHE	Intronic	rs5940465	F8A1	1.07E-79
PRELID1,RAB24	Upstream	rs6879874	PRELID1	3.63E-11
TMX4	UTR5	rs2205783	TMX4	4.98E-17
ZNF19	Exonic	rs8050871	RP11-510M2.2	1.00E-05
MFAP1	Intronic	rs693919	STRCP1	0.000182
S1PR5	Intronic	rs10416073	ICAM3	0.000519
SNRNP70	UTR5	rs11539822	SNRNP70	1.58E-21
CLDN34	Exonic	rs5934730	WWC3	1.92E-05
GOLGA6L4,LOC103171574	Intergenic	rs1808567	DNM1P51	4.94E-09
BIVM,BIVM-ERCC5	Intronic	rs3825519	BIVM	4.17E-17
LIFR	Intronic	rs2256595	LIFR	2.95E-07
CASP1P2,CARD17	Intergenic	rs487128	CASP1P2	3.45E-11
SMG8	Exonic	rs6503905	SMG8	5.60E-14

#### BIVM rs3825519 mutation was identified in BPD patients with prolonged assisted ventilation

BIVM rs3825519 mutation was found in fifteen BPD patients (44.1%, 15/34) and four non-BPD infants (12.5%,4/32), which had statistical difference (*P* = 0.006). We compared the GA, BW, and assisted ventilation duration between the BIVM rs3825519 mutation group (*n* = 15) and the wild-type group (*n* = 19) in BPD patients and found that BIVM mutation group had a longer assisted ventilation duration than the wild-type group (*P* = 0.02, 88.4 ± 49.8 days vs 54.5 ± 26.6 days). In the non-BPD group, the duration of assisted ventilation in the mutant was longer than in wild-type preterm infants (38.5 (29) days vs. 29 (18.5) days), but did not reach statistical difference, may be the sample size need to be expanded. BIVM rs3825519 mutation could lead to aggravation and prolong assisted ventilation of BPD patients, which further confirmed that BIVM gene could play a role in BPD (Table 5).

**Table 5:** The characteristic of patients with BIVM mutation in different groups

Characteristic	BPD group		*P* value	Non-BPD group		*P* value
	Wild-type group (*n* = 19)	BIVM mutation group (*n* = 15)		Wild-type group (*n* = 28)	BIVM mutation group (*n* = 4)	
Gestational age (week)	28.1 ± 2.2	26.9 ± 1.6	0.08	28.9 (2.93)	27.9 (1.25)	0.25
Birth weight (g)	991.1 ± 231.1	894.7 ± 133.5	0.17	1200 (447.5)	1095 (327.5)	0.78
assisted ventilation duration (days, median, IQR)	54.5 ± 26.6	88.4 ± 49.8	0.02	29 (18.5)	38.5 (29)	0.12

Approximately normally distributed data were described using mean ± standard deviation (SD), non-normally distributed data were described using medians and interquartile range (IQR) and non-BPD group with BIVM mutation was non-normally distributed data.

## Discussion

Even though BPD is a common lung disease in premature infants, much remains unknown about the pathogenesis of BPD. Many reports have explored the role of SNPs in BPD, but the reproducibility of the results is poor, suggesting that the genetic variation in BPD may point to uncommon variants and complex genetic factors. In this study, we collected BPD and non-BPD infants for WES analysis. There was no difference in population composition between those two groups. Low GA and BW preterm infants are more likely to develop BPD, which is consistent with currently recognized risk factors. Gender and multiple births are not risk factors for BPD.

We found 457 deleterious SNPs, among them, substitution mutations were the most, followed by deletion mutations, insertion mutations were the fewest, and most of the variants were heterozygous, which was consistent with previous study [9]. One study found BPD patients had more haploinsufficient genes than all protein-coding genes in the human genome [7] and in our study also supported the hypothesis that BPD is dose sensitive to gene, which means more heterozygous mutations may increase the phenotypic advantage of BPD.

Fifty genes in this study had previously been studied in relation to BPD, among which were 6 core genes. However, the mutations carried by the core genes (IL6, EGFR, MMP9, CD44, SERPINE1) were more common in non-BPD, suggesting that these SNPs were not risk factors of BPD. Among them, IL6 rs2069832 is not associated with the incidence of BPD in Northern Ireland and Canadian populations [15], while this locus may have protective significance in this study, reducing the incidence of BPD disease. We found that SOD2 (rs5746091, rs5746090, rs28662077) was only carried in BPD patients and associated with higher GA and BW. Previous study [16] found other two SOD2 SNPs (rs4880 and rs5746136) were associated with lower GA and BW, but not related to the pathogenesis of BPD, contrary to this study. More research is needed to determine whether different mutations in SOD2 gene will make different functions of protein, thus has different influence on BPD. We also identified a novel DDAH1 mutation (NM_012137: exon1: c.89 T > G: p.L30R) in exon region, which is a non-synonymous and deleterious mutation affecting the properties and structure of proteins. Downregulation or reduced activity of DDAH1 leads to apoptotic activation and reduced angiogenesis [17], and rs480414 SNP in DDAH1 was previously found to be protective against the development of pulmonary hypertension in BPD patients [18]. Whether this new mutation affects the activity of DDAH1 and thus plays a pathogenic role deserves further study.

We found 35 genes in our study have been reported in previous WES studies, suggesting they are strongly associated with BPD. Among them, APOB gene was candidate gene in three studies, indicating a possible role in pathogenesis of BPD. Previous study found APOB gene variation associated with the survival rate of non-small cell lung cancer [19], but no research reported the correlation between APOB gene and BPD, which is worth digging into. There were only three overlapping genes between this study and the study of Shanghai [10], considering the differences in the study design, analysis strategy, and definition of BPD may lead to the different results. In this study, the diagnostic definition [12] in 2018 was used, and the definition of BPD in Shanghai used the 2001 [20] criteria. Those genes in our study that overlap with other studies were mainly involved in cellular components, immune-related, microtubule and ciliary organization, angiogenesis, and fibroblasts. On the other hand, we found that the genes associated with BPD disease are mainly concentrated on blood vessels, epithelial cells, fibers, and energy metabolism. This indicates that vascular development, epithelial cell development, collagen fiber, and energy metabolism are closely related to this disease.

We identified 24 candidate genes of BPD, supporting the hypothesis that BPD is a polygenic co-pathogenicity. These genes mainly are related to ciliary movement, and no association with BPD was reported. Through the GEO dataset, we found only BIVM gene expression was decreased in BPD patients, suggesting that BIVM gene is closely associated with BPD. BIMV gene was first identified in the variable region of immunoglobulin gene using electronic search technology, located in the 32–33 region of the long arm of 13 human chromosome, and predicted to encode a 503 amino acid protein, which is ubiquitously expressed in normal tissues and may have immunoglobulin-like functions [21]. Study found BIVM is expressed at the base of cilia and is a key gene in ciliopathies [22]. This study found that BPD patients with BIVM gene mutations needed longer assisted ventilation, which is a high-risk factor of BPD [23]. Therefore, we speculate that the BIVM rs3825519 mutation may affect the function of cilia and be involved in pathogenesis of BPD.

Our report has shortcomings, such as needing a separate sample to validate the results; thus, we plan to further verify the mutation and expression of the candidate genes in another cohort. Despite these limitations, in this study, the use of WES to explore genetic variation of BPD can increase understanding of genetic factors of BPD in China, to guide clinical prediction and intervention, and achieve individualized treatment of BPD.

## Conclusion

For the first time, we identified the role of susceptible SNPs in BPD in Shenzhen, China, and identified 24 candidate genes that could influence the pathogenesis of BPD. And, we also found 35 genes in our study have been reported in previous WES studies, suggesting they have possible roles in pathogenesis of BPD, such as APOB gene. We found BIVM rs3825519 mutation may play a role in the pathogenesis of BPD by affecting ciliary motility. A novel DDAH1 mutation site (NM_012137: exon1: C.89 T&GT; G: P.L30r) may be involved in the pathogenesis of BPD.

## Supplementary Information

Below is the link to the electronic supplementary material.

Supplementary file1 (XLSX 25 KB)

Supplementary file2 (DOCX 17 KB)

## Data Availability

All data relevant to the study are included in the article and its online supplementary material.

## Abbreviations

APOB: Apolipoprotein B
BIVM: For basic, immunoglobulin-like variable motif-containing
BW: Birth weight
DDAH1: Dimethylarginine dimethylaminohydrolase 1
GA: Gestational age
SNP: Single-nucleotide polymorphism
SOD2: Superoxide dismutase 2
WES: Whole-exon sequencing
