# Supplementary material for: Identification of genetic susceptibility in preterm newborns with bronchopulmonary dysplasia by whole-exome sequencing: BIVM gene may play a role
Source: Eur J Pediatr. 2023 Feb 9;182(4):1707–18. doi: 10.1007/s00431-022-04779-z (PMC10167099; doi:10.1007/s00431-022-04779-z)
Supplement: Supplementary file 2 — Supplementary file2 (DOCX 17 KB) [file 431_2022_4779_MOESM2_ESM.docx]

**Supplementary table 2. The SNPs in IL6, EGFR, MMP9, CD44, SERPINE1 and TLR4 genes were not risk factors of BPD in our study**

| gene | rs | non-BPD | BPD | Vary type | region | P value |
| --- | --- | --- | --- | --- | --- | --- |
| IL6 | rs2069832 | 20(62.5%) | 11(32.4%) | substitution | intronic | 0.026 |
|  | rs1474347 | 24(75%) | 16(47.1%) | substitution | intronic | 0.025 |
| EGFR | rs11506105 | 23(71.9%) | 16(47.1%) | substitution | intronic | 0.049 |
|  | rs17336919 | 19(59.4%) | 10(29.4%) | substitution | intronic | 0.025 |
| MMP9 | rs3787268 | 17(53.1%) | 8(23.5%) | substitution | intronic | 0.022 |
| CD44 | rs76393888 | 10(31.3%) | 3(8.8%) | substitution | intronic | 0.031 |
|  | rs3215691 | 10(31.3%) | 3(8.8%) | deletion | intronic | 0.031 |
|  | rs34986068 | 23(71.9%) | 14(41.2%) | deletion | intronic | 0.015 |
| SERPINE1 | rs2227692 | 13(40.6%) | 5(14.7%) | substitution | intronic | 0.027 |
|  | rs2854236 | 10(31.3%) | 3(8.8%) | substitution | intronic | 0.031 |
